# Low-Complexity and Hardware-Friendly H.265/HEVC Encoder for Vehicular Ad-Hoc Networks

**DOI:** 10.3390/s19081927

**Published:** 2019-04-24

**Authors:** Xiantao Jiang, Jie Feng, Tian Song, Takafumi Katayama

**Affiliations:** 1 Department of Information Engineering, Shanghai Maritime University, Shanghai 201306, China; 2 State Key Laboratory of Integrated Services Networks, Department of Telecommunications Engineering, Xidian University, Xi’an 710071, China; jiefengcl@163.com; 3 Department of Electrical and Electronics Engineering, Tokushima University, 2-24, Shinkura-cho, Tokushima 770-8501, Japan; tiansong@ee.tokushima-u.ac.jp (T.S.); katayama@ee.tokushima-u.ac.jp (T.K.)

**Keywords:** high efficiency video coding, low complexity, hardware friendly, vehicular ad-hoc networks

## Abstract

Real-time video streaming over vehicular ad-hoc networks (VANETs) has been considered as a critical challenge for road safety applications. The purpose of this paper is to reduce the computation complexity of high efficiency video coding (HEVC) encoder for VANETs. Based on a novel spatiotemporal neighborhood set, firstly the coding tree unit depth decision algorithm is presented by controlling the depth search range. Secondly, a Bayesian classifier is used for the prediction unit decision for inter-prediction, and prior probability value is calculated by Gibbs Random Field model. Simulation results show that the overall algorithm can significantly reduce encoding time with a reasonably low loss in encoding efficiency. Compared to HEVC reference software HM16.0, the encoding time is reduced by up to 63.96%, while the Bjontegaard delta bit-rate is increased by only 0.76–0.80% on average. Moreover, the proposed HEVC encoder is low-complexity and hardware-friendly for video codecs that reside on mobile vehicles for VANETs.

## 1. Introduction

Vehicular ad-hoc networks (VANETs) can provide multimedia communication between vehicles with the aim of providing efficient and safe transportation [1]. Vehicles with different sensors can exchange and share information for safely breaking, localization and obstacle avoiding. Moreover, the sharing of traffic accident’s live video can improve the rescue efficiency and alleviate traffic jams. However, video transmission has been considered as a challenging task for VANETs, because video transmission over VANETs can significantly increase bandwidth [2]. This work focuses on the development of video codec that supports real-time video transmission over VANETs for road safety applications.

The demanding challenges of VANETs are bandwidth limitations and opportunities, connectivity, mobility, and high loss rates [3]. Because of the resource-demanding nature of video data in road safety applications, bandwidth limitations is the bottleneck for real-time video transmission over VANETs [4,5]. Moreover, due to the limited vehicle node’s battery lifetime, video delivery over VANETs remains extremely challenging. Essentially, the low-complexity video encoder can accelerate the video transmission in real-time, and achieve low delay for video streaming. Hence, in order to transmit real-time video with bandwidth constraint, it is vital to develop an efficient video encoder. A video encoder with high encoding efficiency and low encoding complexity is the core requirement of VANETs [6].

Concurrently, the main video codecs are High Efficiency Video Coding (HEVC, or H.265) that are developed by the Joint Collaborative Team on Video Coding (JCT-VC) Group, and AV1 that is developed by Alliance for Open Media (AOMedia) [7]. Nevertheless, video codec is faced with several challenges. AV1 is a newer codec, royalty free and open sourced. However, the hardware implementation of AV1 encoder will take a long time. H.265/HEVC is the state-of-the-art standardized video codecs [8]. Compared with H.265/HEVC, AV1 increases the complexity significantly without achieving an increase in coding efficiency. When supporting the most available (resource-limited/mobile) devices or having a need for real-time, low latency encoding, it would be better to stick to H.265/HEVC. However, the encoding complexity of H.265/HEVC encoder increases dramatically due to its recursive quadtree representation [9]. Although previous excellent works have been proposed for reducing H.265/HEVC encoder complexity [10,11,12,13,14], most of them balance the encoding complexity and encoding efficiency unsuccessfully.

To address this issue, spatial and temporal information is widely used to reduce the computation redundancy of the H.265/HEVC encoder. However, the spatiotemporal correlation between the coding unit and the neighboring coding unit is not better used. To the author’s best knowledge, complexity reduction in real-time coding with the available computational power at the VANET nodes has not been well studied, especially from the viewpoint of hardware implementation. The key contributions of this work are summarized as follows:

- We propose a low-complexity and hardware-friendly H.265/HEVC encoder. The proposed encoder allows the encoding complexity to be reduced significantly so that low delay requirements for video transmission in power-limited VANETs nodes are satisfied.
- A novel spatiotemporal neighboring set is used to predict the depth range of the current coding tree unit. The prior probability of coding unit splitting or non-splitting is calculated with the spatiotemporal neighboring set. Moreover, the Bayesian rule and Gibbs Random Field (GRF) are used to reduce the encoding complexity for H.265/HEVC encoder with the combination of the coding tree unit depth decision and prediction unit modes decision.
- The proposed algorithm can balance the encoding complexity and encoding efficiency successfully. The encoding time can be reduced by 50% with negligible encoding efficiency loss, and the proposed encoder is suitable for real-time video applications.

The rest of paper is organized as follows. Related works are reviewed in Section 2. Section 3 discusses background details. In Section 4, the fast CU decision algorithm is presented. Simulation results are discussed in Section 5. Section 6 concludes this work.

## 2. Related Work

### 2.1. Video Streaming in Vehicular Ad-Hoc Networks

Real-time Video transmission over VANETs can improve emergency responses’ effectiveness for road safety applications. The high rates and low delay are the most challenging aspects of video transmission over VANETs [15]. Recently, some works have been proposed to solve this problem. Different video applications over VANETs need different resources. The collaborative vehicle to vehicle communication approach is presented to enhance the scalable video quality in intelligent transportation systems (ITS), and different methods are developed to enhance the quality of experience and quality of service during scalable video transmission over VANETs [16]. Meanwhile, the use of redundancy for video streaming over VANETs has been analyzed, and a selective additional redundancy approach is proposed to improve the video quality [17]. Moreover, in Ref. [18], a vehicle rewarding method for video transmission over VANETs using real neighborhood and relative velocity is presented to optimize video transmission. Although previous works have studied the issues of video streaming over VANETs, the performance of video codec to support real-time video transmission is missing.

### 2.2. Low Complexity Algorithm for H.265/HEVC Encoder

To reduce the encoding complexity of the H.265/HEVC encoder, some fast algorithm of coding unit (CU) size decision and prediction unit (PU) mode decision is presented. In previous works, the main spatiotemporal parameters that are used for fast CU size decisions include the neighboring CU depth, rate-distortion (RD) cost, motion vector (MV), coded block flag (cbf), and sample-adaptive-offset (SAO) information. Moreover, some other statistical learning based CU selection methods are proposed include Bayesian classifier, support vector machine (SVM), decision tree (DT), AdaBoost classifier and artificial neural network (ANN).

Jiang et al. presented a fast encoding complexity method based on the probabilistic graphical model [19,20]. These proposed algorithms consist of CU early termination and CU early skip methods to reduce the redundant computing of inter-prediction in H.265/HEVC. However, these methods cannot achieve better trade-off between the encoding efficiency and encoding complexity. Refs. [21,22] focused on decreasing the CU depth to reduce the encoding complexity of the H.265/HEVC encoder. In Ref. [23], the unimodal stopping model-based early skip mode decision was used to speed up the process of mode decision. This proposed early skip mode decision method can reduce encoding time significantly. In Ref. [24], a fast algorithm for the H.265/HEVC encoder was based on the Markov Chain Monte Carlo (MCMC) model and Bayesian classifier. Even though the above fast CU size decision methods utilized the spatiotemporal correlations, the fast PU mode decision methods are ignored.

Tai et al. introduced three novel methods including early CU split, early CU termination and search range adjustment to reduce the computation complexity for H.265/HEVC [25]. This proposed algorithm can outperform previous works with respect to both the speed and the RD performance. In Ref. [26], a fast inter CU decision was proposed based on the latent sum of absolute differences (SAD) estimation. This proposed algorithm achieved an average of 52% and 58.4% reductions of the encoding time. Refs. [27,28] focused on CU size decision and PU mode decision, the fast encoding algorithms based on statistical analysis were proposed to reduce the encoding complexity for the H.265/HEVC encoder. This method can reduce about 57% and 55% of the encoding time of the H.265/HEVC encoder. The above methods can significantly reduce the encoding complexity with the joining of the CU depth and PU modes prediction, however, these previous works cannot balance the encoding complexity and encoding efficiency successfully. Moreover, the cost of hardware implementation is higher for previous works.

All in all, this paper focus on the development of video encoder that supports real-time video streaming over VANETs. We design a low-complexity and hardware-friendly encoder to allow video transmission to adapt to the VANETs environment. In addition, compared with the current literature, the proposed encoder can achieve better performance trade-off.

## 3. Technical Background

### H.265/HEVC

**Figure 1:**
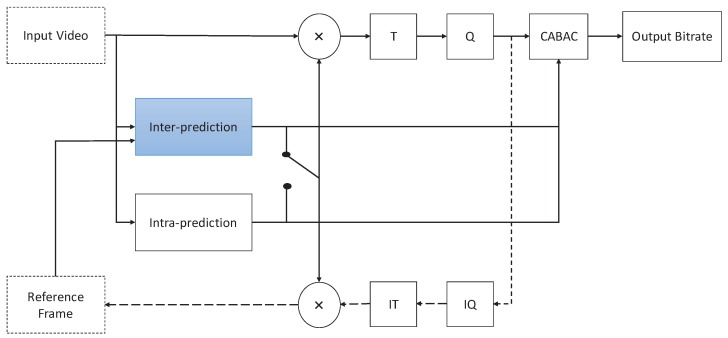
The structure of high efficiency video coding (HEVC, or H.265) encoder.

H.265/HEVC standard was released in 2013 by JCT-VC, which can reduce bit-rates by about 50% over H.264. In addition, H.265/HEVC adopts hybrid video compression technology, and the typical structure of H.265/HEVC encoder is shown in Figure 1. The main modules of H.265/HEVC encoder include: (1) Intra-prediction and inter-prediction, (2) transform (T), (3) quantization (Q) and (4) context-adaptive binary arithmetic coding (CABAC) entropy coding. Moreover, inter-prediction and intra-prediction modules are used to decrease the spatial and temporal redundancy. Transform and quantization modules are used to decrease visual redundancy. The entropy coding module is used to decrease the information entropy redundancy. It is noted that the inter-prediction module is the most critical tool, which consumes about 50% computation complexity. Then, in order to achieve real-time coding, the computation complexity of H.265/HEVC encoder should be reduced by decreasing spatiotemporal redundancy.

**Figure 2:**
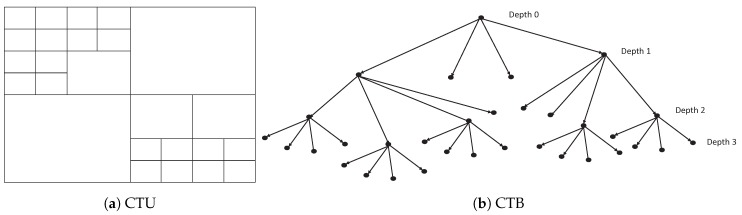
Coding tree unit (CTU) partitioning and coding tree block (CTB) structure.

The video frame is divided into a lot of coding tree units (CTUs) in H.265/HEVC standard. A CTU includes a coding tree block (CTB) of the luma samples, two CTBs of the chroma samples, and associated syntax elements. The CTU size can be adjusted from 16×16 to 64×64. Each CTU can be divided into four square CUs, and a CU can be recursively divided into four smaller CUs. A CU consists of a coding block (CB) of the luma samples, two CBs of the choma samples, and the associated syntax elements. The CU size can be 8×8, 16×16, 32×32, or 64×64. Figure 2 shows an example of the CTB structure for a given CTU. The CTU in Figure 2a is divided into different sized CUs. Correspondingly, the CTB structure is shown in Figure 2b. In each depth of CTB, the rate-distortion (RD) cost of each node is checked until the RD cost is minimum.

**Figure 3:**
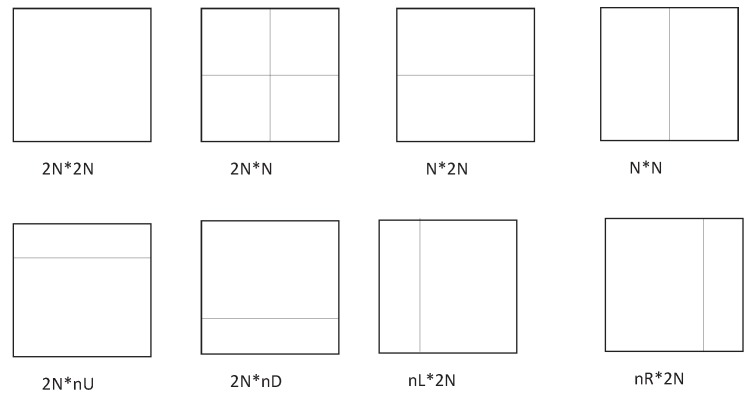
PU modes in H.265/HEVC inter-prediction.

The prediction unit (PU) can be transmitted in the bitstream, which identifies the prediction mode of CU. A PU consists of a prediction block (PB) of the luma, two PB of the chroma, and associated syntax elements. Figure 3 shows the eight partition modes that may be used to define the PUs for a CU in H.265/HEVC inter-prediction. For a CU configured to use inter-prediction, all eight partitions include four symmetry modes (2N*2N, 2N*N, N*2N, N*N) and four asymmetric modes (2N*nU, 2N*nD, nL*2N, nR*2N).

A CU can be recursively divided into transform units (TUs) according to the quadtree structure, and CU is the root of the quadtree. The TU is a basic representative block having residual or transform coefficients. In TU, one syntax element named coded block flag (cbf) indicates whether at least one non-zero transform coefficient is transmitted for the whole CU. When there is a non-zero coefficient, cbf is equal to 0. When there is no non-zero coefficients, cbf is equal to 1. Moreover, cbf is an important factor for the CU size decision [14].

The advantage of block partitioning structure is that the arbitrary size of CTU enables the codec to be readily optimized for various contents, applications, and devices. However, the recursive structure of coding block causes lots of redundant computing. In order to support the real-time video transmission over VANETs, the redundant computing of the H.265/HEVC encoder should be decreased significantly.

## 4. The Proposed Low-Complexity and Hardware-Friendly H.265/HEVC Encoder for VANETs

### 4.1. The Novel Spatiotemporal Neighborhood Set

The object motion is regular in video sequences and there is some continuity in the depth between adjacent CUs. If the depth range of the current CU can be inferred from the encoded neighboring CU, then some hierarchical partitioning is directly skipped or terminated. Therefore, the computational complexity has been reduced, significantly.

In order to utilize the spatiotemporal correlation, the four neighborhood set G is defined as

(1) $$\begin{array}{c} G=\{CU_{L},CU_{TL},CU_{TR},CU_{CO}\}. \end{array}$$

**Figure 4:**
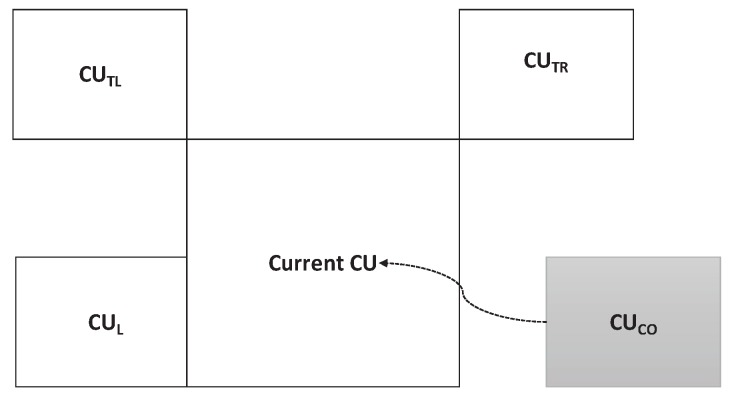
Spatiotemporal neighborhood set.

Set *G* is shown in Figure 4, where $CU_{L}$, $CU_{TL}$, $CU_{TR}$, and $CU_{CO}$ denote the left, top-left CU, top-right, and collocated of the current CU, respectively.

### 4.2. CTU Depth Decision

For video compression techniques, a smooth coding block popularly has the smaller CU depth. By contrast, the larger depth value is suitable for a complex area. Previous works show that the object motion in the same frame remains directional, and the motion and texture of the neighboring CUs are similar. In this work, the depths of neighboring CTU in the set *G* are used to predict the depth range of current CTU, and the predicted depth of current CTU is calculated as

(2) $$\begin{array}{c} {\hat{Dep}}_{CTU}=\sum_{k=0}^{3}\theta_{k}\times Dep_{k}, \end{array}$$

where *k* is the index of neighboring CTU in set *G*, $Dep_{k}$ is the depth of neighboring CTU in the set *G*, and $\theta_{k}$ is a weight factor of neighboring CTU’s depth, respectively, in the set *G*. In the H.265/HEVC standard, the range of CTU is depth 0, 1, 2, and 3. Hence, the calculated depth of the current CTU (${\hat{Dep}}_{CTU}$) satisfies

(3) $$\begin{array}{c} {\hat{Dep}}_{CTU}\leq 3. \end{array}$$

In Equation (3), $Dep_{k}\leq 3$. Therefore, weight factor $\theta_{k}$ satisfies

(4) $$\begin{array}{c} \sum_{i=0}^{3}\theta_{k}\leq 1. \end{array}$$

If the range of current CTU is depth 0, 1, 2, and 3, then the sum of weight factor $\theta_{k}$ is 1 in this work. Moreover, Zhang’s work confirms that, when the weight factor of the spatial neighboring CTU’s depth is more than the weight factor of the temporal neighboring CTU’s depth, the calculated CTU depth is closer to the actual depth of the current CTU [29]. In this work, each weight factor of the spatial neighboring CTU’s depth is equal, and the weight factor of spatial neighboring CTU’s depth is more than the weight factor of temporal CTU’s depth. Then $\theta_{k}$ satisfies

(5) $$\theta_{k}=\left\{\begin{array}{cc} 0.3, & \mathrm{if}\;k\;=\;0,\;1,\;2\\ 0.1, & \mathrm{if}\;k\;=\;3\; \end{array}.\right.$$

However, the calculated value of ${\hat{Dep}}_{CTU}$ is a non-integer most of the time. It is not suitable to directly predict the depth of current CTU by the value of ${\hat{Dep}}_{CTU}$. Therefore, the rule of CTU depth range has been formulated as Table 1, and the depth range of current CTU can be generated with the value of ${\hat{Dep}}_{CTU}$.

Due to the predicted depth of the current CTU, each CTU can be divided into three types: T1,T2,T3. The CTU depth range can be decided from Table 1. The expressions of the relation between CTU type, ${\hat{Dep}}_{CTU}$, and CTU depth are as follows.

(1) when the predicted depth of current CTU ${\hat{Dep}}_{CTU}$ satisfies ${\hat{Dep}}_{CTU}$ ≤ 1.5, it means that the motions of neighboring CTUs are smooth and the depths of neighboring CTUs are small. The current CTU belongs to the still or homogeneous motion region and is classified as type T1. In this case, the minimum depth of current CTU $Dep_{min}$ is equal to “0”, and the maximum depth of current CTU $Dep_{max}$ is equal to “2”.
(2) when the predicted depth of current CTU ${\hat{Dep}}_{CTU}$ satisfies 1.5 < ${\hat{Dep}}_{CTU}$ ≤ 2.5, it means that the depths of neighboring CTUs are middle. The current CTU belongs to the moderate motion region and is classified as type T2. In this case, the minimum depth of current CTU $Dep_{min}$ is equal to “1”, and the maximum depth of current CTU $Dep_{max}$ is equal to “3”.
(3) when the predicted depth of current CTU ${\hat{Dep}}_{CTU}$ satisfies 2.5 < ${\hat{Dep}}_{CTU}$ ≤ 3, it means that the motions of neighboring CTUs are intense and the depths of neighboring CTUs are high. The current CTU belongs to the fast motion region and is classified as type T3.

In this case, the minimum depth of current CTU $Dep_{min}$ is equal to “2”, and the maximum depth of current CTU $Dep_{max}$ is equal to “3”.

### 4.3. PU Mode Decision

The CU splitting or non-splitting is formulated as a binary classification problem $\omega_{i}$, where *i* = 0, 1. In this work, $\omega_{0}$ and $\omega_{1}$ respectively represent CU non-splitting and CU splitting, and the variable *x* represents the RD-cost of the PU. According to the Bayes’ rule, the posterior probability $p(\omega_{i}|x)$ can be calculated as follows:

(6) $$\begin{array}{c} p\left(\omega_{i}|x\right)=\frac{p\left(x|\omega_{i}\right)p\left(\omega_{i}\right)}{p(x)}. \end{array}$$

According to Bayesian decision theory, the prior probability $p(\omega_{i})$ and the conditional probability $p(x|\omega_{i})$ values must be known. Therefore, CU non-splitting ($\omega_{0}$) will be chosen if the following condition holds true:

(7) $$\begin{array}{c} p\left(\omega_{0}|x\right)>p\left(\omega_{1}|x\right). \end{array}$$

Otherwise, CU splitting ($\omega_{1}$) will be chosen.

The conditional probability $p(x|\omega_{0})$ and $p(x|\omega_{1})$ are the probability density function of the RD cost, and they are approximated by normal distributions. Defining the mean values and covariance of RD cost of CU non-splitting and splitting as $N(\mu_{0},\sigma_{0})$ and $N(\mu_{1},\sigma_{1})$, the normal function can be given by

(8) $$\begin{array}{c} p\left(x|\omega_{0}\right)=\frac{1}{\sqrt{2\pi }\sigma_{0}}exp\left\{-\frac{{\left(x-\mu_{0}\right)}^{2}}{2{\sigma_{0}}^{2}}\right\},\;p\left(x|\omega_{1}\right)=\frac{1}{\sqrt{2\pi }\sigma_{1}}exp\left\{-\frac{{\left(x-\mu_{1}\right)}^{2}}{2{\sigma_{1}}^{2}}\right\}. \end{array}$$

The prior probability $p(\omega_{i})$ is modeled with Gibbs Random Fields (GRF) model in set *G* [30], and $p(\omega_{i})$ will always have the Gibbsian form

(9) $$\begin{array}{c} p\left(\omega_{i}\right)=Z^{-1}exp\left(-E\left(\omega_{i}\right)\right),\;E\left(\omega_{i}\right)=\sum_{k\in G}\varphi \left(\omega_{i},{\bar{\omega }}_{k}\right). \end{array}$$

where *Z* is a normalization constant, and $E(\omega_{i})$ is cost function. *k* is the index of set *G*, and ${\bar{\omega }}_{k}$ denotes the non-splitting or splitting value of the neighborhood *k*-CU (${\bar{\omega }}_{k}=-1,1$). The CU size decision deals with the binary classification problem ($\omega_{i}=-1,1$), and the clique potential $\varphi (\omega_{i},{\bar{\omega }}_{k})$ obeys the Ising model [31]:

(10) $$\begin{array}{c} \varphi \left(\omega_{i},{\bar{\omega }}_{k}\right)=-\gamma \times \left(\omega_{i}\times {\bar{\omega }}_{k}\right), \end{array}$$

where the parameter γ is the coupling factor, which denotes the strength of current CU correlation with neighborhood *k*-CU in set *G*. In this work, γ is set to “0.75”. Then, the prior $p(\omega_{i})$ can be written in the factorized form:

(11) $$\begin{array}{c} p\left(\omega_{i}\right)\propto exp\left(-E\left(\omega_{i}\right)\right)=exp\left(\sum_{k\in G}-\gamma \times \left(\omega_{i}\times {\bar{\omega }}_{k}\right)\right). \end{array}$$

At last, the Equation (6) can be written as

(12) $$\begin{array}{c} p\left(\omega_{i}|x\right)\propto p\left(x|\omega_{i}\right)p\left(\omega_{i}\right)\propto exp\left(\sum_{k\in G}-\gamma \times \left(\omega_{i}\times {\bar{\omega }}_{k}\right)\right)\times \frac{1}{\sigma_{i}}exp\left\{-\frac{{\left(x-\mu_{i}\right)}^{2}}{2{\sigma_{i}}^{2}}\right\}. \end{array}$$

Finally we can define the final CU decision function as $S(\omega_{i})$, which can be written in the exponential form

(13) $$S\left(\omega_{i}\right)=exp\left(\sum_{k\in G}-\gamma \times \left(\omega_{i}\times {\bar{\omega }}_{k}\right)\right)\times \frac{1}{\sigma_{i}}exp\left\{-\frac{{\left(x-\mu_{i}\right)}^{2}}{2{\sigma_{i}}^{2}}\right\}.$$

**Figure 5:**
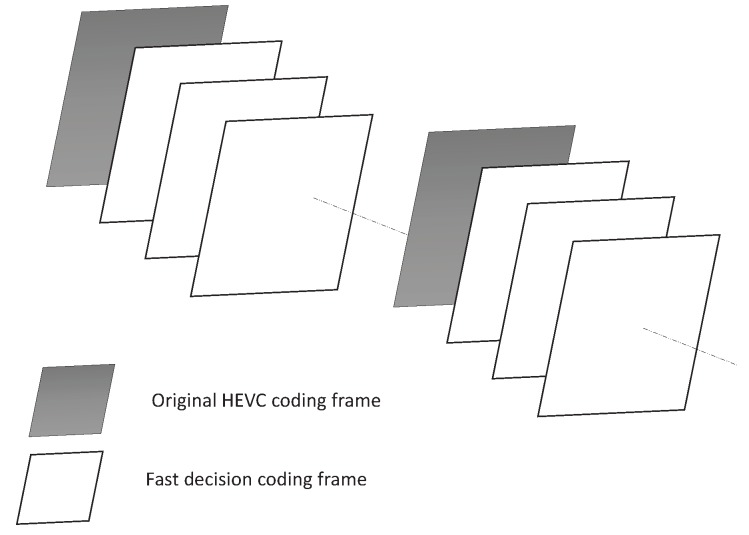
The statistical parameters are estimated with online learning.

It should be noted that the statistical parameters $p(x|\omega_{i})$ are estimated by using a non-parametric estimation with online learning, and are stored in a lookup table (LUT). The frames used for online updating of the values of $\left(\mu_{0},\sigma_{0}\right)$ and $\left(\mu_{1},\sigma_{1}\right)$ are shown as in Figure 5. In each group of pictures(GOP), the 1st frame that can be encoded by using the original H.265/HEVC coding will be used for the online update, while the successive frames are coded by using the proposed algorithm.

Through the above analysis, the proposed PU decision based on Bayes’ rule includes the CU termination decision (inter 2N*2N) and CU skip decision (inter 2N*2N, N*2N, 2N*N). In the case of the CU termination decision, the current CU is not divided into sub-CUs in the sub-depth. In the case of the CU skip decision, the current PU mode in current CU depth is determined at the earliest possible stage. Therefore, the flowchart of the proposed PU mode decision is described as follows.

(1) At the encoding time for inter prediction, first of all, look up the statistical parameters in LUT. Then, the RD cost of the inter 2N*2N PU mode is checked. If the condition satisfies $S(\omega_{0})$ > $S(\omega_{1})$ and cbf = 0, the CU termination decision is processed. Otherwise, if the condition is satisfying $S(\omega_{1})$ > $S(\omega_{0})$ and cbf = 1, a CU skip decision is made.
(2) RD cost of the inter 2N*N PU mode is checked. If the condition is satisfying $S(\omega_{1})$ > $S(\omega_{0})$ and cbf = 1, a CU skip decision is made.
(3) RD cost of the inter N*2N PU mode is checked. If the condition is satisfying $S(\omega_{1})$ > $S(\omega_{0})$ and cbf = 1, a CU skip decision is made.
(4) Other PU modes are checked according to the H.265/HEVC reference model.

### 4.4. The Overall Framework

**Figure 6:**
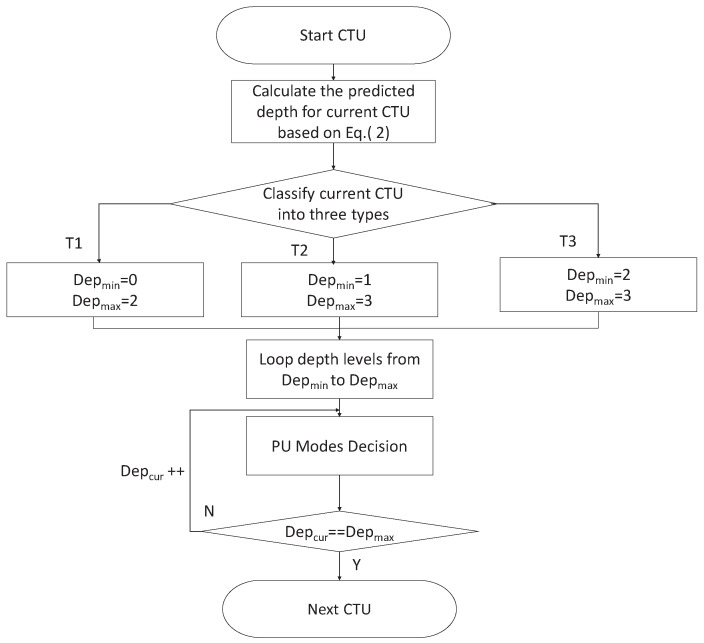
Flowchart of the proposed CTU depth decision.

**Figure 7:**
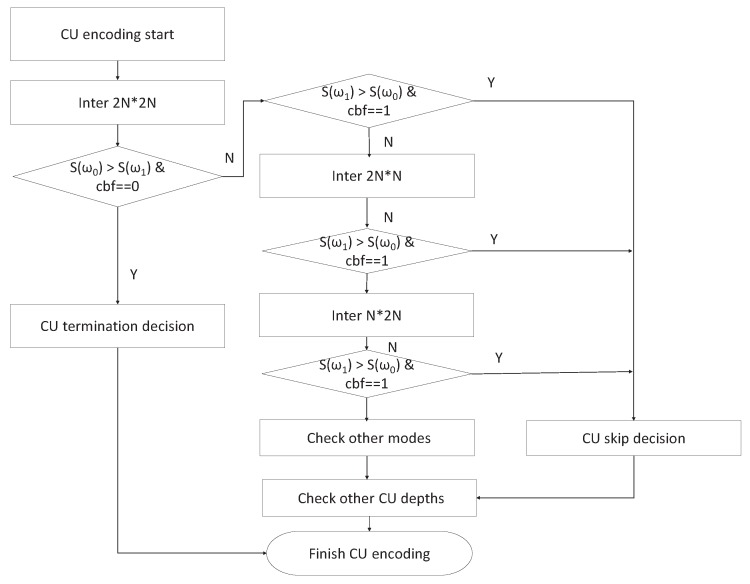
Flowchart of the proposed prediction unit (PU) mode decision.

Based on the above analysis, the proposed overall algorithm incorporates the CTU depth decision and the PU mode decision algorithms to reduce the computation complexity of the H.265/HEVC encoder. The flowcharts are shown in Figure 6 and Figure 7, respectively. The proposed CTU depth decision and PU mode decision algorithms have been discussed in Section 4.2 and Section 4.3.

It is noted that the maximum GOP size is equal to “8” in this work, and the value of $\left(\mu_{0},\sigma_{0}\right)$ and $\left(\mu_{1},\sigma_{1}\right)$ are updated every GOP for PU mode decision.

### 4.5. Encoder Hardware Architecture

**Figure 8:**
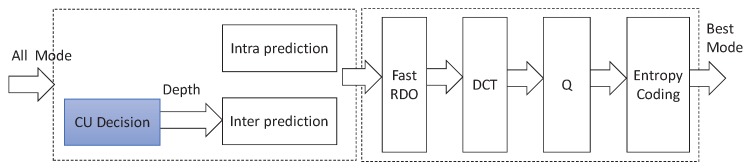
Mode decision process.

Figure 8 shows the core architecture of the H.265/HEVC with mode decision. By using the architecture, inter-frame prediction is used to eliminate the spatiotemporal redundancy. The proposed CU decision method can accelerate the inter-prediction module before fast rate-distortion optimization (RDO). The novel spatiotemporal neighboring set is used to reduce the complexity of inter encoder which leads to a very low-power cost. Moreover, video codec on mobile vehicles for VANETs need to be more energy efficient and more reliable, so reducing the complexity of the video encoder is important. Then, the proposed low-complexity and hardware-friendly H.265/HEVC encoder can ensure the reliability of the video codec for VANETs significantly. Moreover, as a benefit of the high complexity reduction rate, the energy consumption can be reduced for hardware design, significantly.

## 5. Experimental Results

To evaluate the performance of the proposed low-complexity and hardware-friendly H.265/HEVC encoder for VANETs, this section shows the experimental results by implementing the proposed algorithms with the H.265/HEVC reference software [32]. The simulation environments are shown in Table 2.

The Bjontegaard delta bit-rate (BDBR) is used to represent the average bit-rate [33], and the average time saving (TS) is calculated as

(14) $$\begin{array}{c} TS=\frac{1}{4}\times \sum_{i=1}^{4}\frac{Time_{HM16.0}\left(QP_{i}\right)-Time_{proposed}\left(QP_{i}\right)}{Time_{HM16.0}\left(QP_{i}\right)}\times 100\% \end{array}$$

where $Time_{HM16.0}\left(QP_{i}\right)$ and $Time_{proposed}\left(QP_{i}\right)$ denote the encoding time of using HM16.0 and the proposed algorithm with different QP.

**Figure 9:**
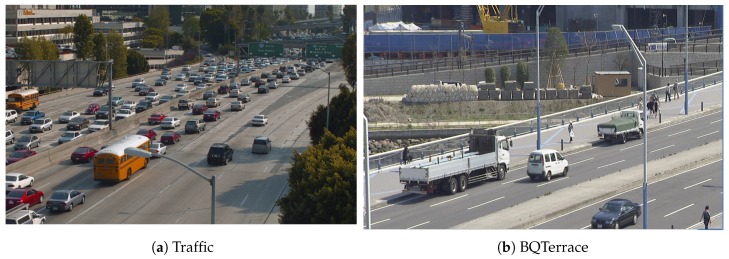
Traffic scenario.

In this work, the scenarios have been chosen carefully. This work focuses on the development of a video codec that supports real-time video transmission over VANETs for road safety applications. The common test conditions (CTC) are provided to conduct experiments [34]. The test sequences in CTC have different spatial and temporal characteristics and frame rates. Furthermore, the video sequences of traffic scenarios including ‘Traffic’ and ‘BQTerrace’ (as in Figure 9) are tested in this work. Moreover, we selected low delay (LD) configuration to reflect the real-time application scenario for all encoders.

Table 3 and Table 4 show the performance results of the CTU depth decision, PU mode decision and the overall (proposed) methods, compared to H.265/HEVC reference software in random access (RA) and low delay (LD) configurations. From the experimental results on Table 3, It can be seen that the encoding time can be reduced by 15.59%, 55.79%, and 50.96% on average for CTU depth decision, PU mode decision, and overall methods, while the BDBR can be incremented by only 0.11%, 0.96%, and 0.80%, respectively. From the experimental results in Table 4, the encoding time can be reduced by 14.05%, 50.28%, and 50.23% on average for CTU depth decision, PU mode decision, and overall methods, while the BDBR can be incremented by only 0.15%, 0.79%, and 0.76%, respectively. For high-resolution of sequences such as “BQTerrace”, and “Vidyo4”, the time saving is particularly high. Therefore, the overall (proposed) algorithm can significantly reduce the encoding complexity and rarely affects encoding efficiency. Moreover, the proposed method can achieve the trade-off between the encoding complexity and the encoding efficiency. In addition, the optimal tradeoff of encoding performance can be adjusted by the coupling factor γ. Therefore, the optimal tradeoff of encoding performance is that the encoding complexity can be reduced significantly with less than or equal to 0.8% encoding efficiency, and less low delay (LD) and random access (RA) configuration. In order to find the optimal tradeoff with coupling factor γ, the γ is set to “0.5”, “0.75” and “0.85” under the same simulation environments. The compared results of the average efficiency and time saving are shown as in Table 5. From this table we can see that, in this case of γ = 0.75, the encoding performance is optimal in this work.

**Figure 10:**
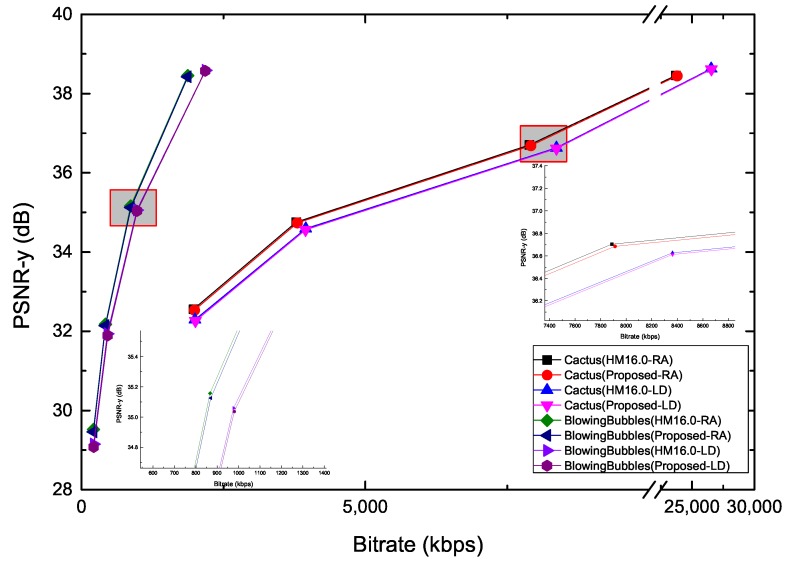
Rate–distortion (R–D) curve of the proposed method for “Cactus” and “BlowingBubbles”.

**Figure 11:**
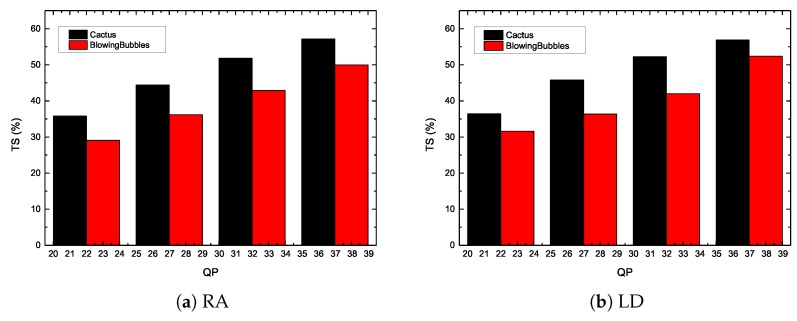
Time savings of the proposed method for “Cactus” and “BlowingBubbles”.

Video objective quality evaluation can be expressed by rate–distortion (R–D) curve. The R–D curve is fitted through four data points, and PSNR/bit-rate are assumed to be obtained for QP = 22, 27, 32, 37. In addition, when an error on the predicted depth of the current CTU occurs, the bit-rate will increase. In this paper, the video objective quality is evaluated by using bit-rate and PSNR. Then the lower the accuracies of the predicted depth of the current CTU algorithm, the more the bit-rate increases. Figure 10 shows the R–D curve of the proposed method, compared with the H.265/HEVC reference software. It can be noticed that the enlarged part of the figure shows the proposed algorithm is close to HM16.0 under the LD and RA configurations. In addition, Figure 11 shows the time saving of the sequences “Cactus” and “BlowingBubbles”. It is noted that the encoding time can be reduced under different configurations.

The performance comparison of the proposed method is shown in Table 6, compared to previous works [12,13,14,24,25,26]. Goswami’s work is based on Bayesian decision theory and Markov Chain Monte Carlo model (MCMC). Zhang’s work is based on the Bayesian method and Conditional Random Fields (CRF). Tai’s algorithm is based on depth information and RD cost. Zhu’s algorithm is based on the machine learning method. Ahn’s work is based on spatiotemporal encoding parameters. Xiong’s work is based on the latent sum of absolute differences (SAD) estimation. However, the proposed approach is based on Bayesian rule and Gibbs Random Field. Although Zhu’s method can achieve a 65.60% encoding time reduction, the BDBR is higher than the proposed method. Moreover, the increasing of the BDBR is smaller than state-of-the-art works, while the time saving is more than 50% on average. Compared with previous works [19,20], the proposed work can trade-off the encoding complexity and encoding efficiency successfully.

## 6. Conclusions

In order to develop the low-complexity and hardware-friendly H.265/HEVC encoder for VANETs, based on a novel spatiotemporal neighborhood set, the Bayesian rule and Gibbs Random Field are used to reduce the encoding complexity for the H.265/HEVC inter-prediction in this work. The proposed algorithm consists of CTU depth decision and PU mode decision methods. Experimental results demonstrate that the proposed approach can reduce the average encoding complexity of H.265/HEVC encoder by about 50% for VANETs, while the increasing of BDBR is less than or equal to 0.8% on average.

## Figures and Tables

**Table 1:** The CTU depth range.

CTU Type	${\hat{\mathrm{Dep}}}_{\mathrm{CTU}}$ Range	The CTU Depth Range
T1	${\hat{Dep}}_{CTU}$ ≤ 1.5	[0, 1, 2]
T2	1.5 < ${\hat{Dep}}_{CTU}$ ≤ 2.5	[1, 2, 3]
T3	2.5 < ${\hat{Dep}}_{CTU}$ ≤ 3	[2, 3]

**Table 2:** The simulation environments.

Items	Descriptions
Software	HM16.0
Video Size	2560×1600, 1920×1080, 1280×720, 832×480, 416×240
Configurations	random access (RA), low delay (LD)
Quantization Parameter (QP)	22, 27, 32, 37
Maximum CTU size	64×64

**Table 3:** Performance comparison of different parts of the proposed method (random access (RA)).

		CTU Depth Decision		PU Mode Decision		Overall (Proposed)	
Size	Sequence	BDBR(%)	TS(%)	BDBR(%)	TS(%)	BDBR(%)	TS(%)
2560×1600	Traffic	0.19	12.46	1.15	58.03	1.00	52.31
	SteamLocomotive	0.12	13.79	0.82	56.32	0.72	52.65
1920×1080	ParkScene	0.14	12.83	1.03	56.93	0.83	51.76
	Cactus	0.13	12.25	1.34	52.57	1.19	47.31
	BQTerrace	0.02	14.18	0.84	57.54	0.69	54.09
832×480	BasketballDrill	−0.13	14.11	0.73	51.55	0.50	46.10
	BQMall	0.18	15.97	0.92	56.76	0.73	51.37
	PartyScene	0.06	17.34	0.75	50.09	0.61	44.96
	RaceHorses	0.02	13.59	1.31	44.27	1.08	37.10
416×240	BasketballPass	0.26	7.09	0.90	54.73	0.60	46.40
	BQSquare	0.05	14.37	0.57	54.08	0.44	45.93
	BlowingBubbles	0.17	8.54	1.26	48.12	1.09	39.53
1280×720	Vidyo1	0.11	16.19	1.18	66.60	0.77	63.30
	Vidyo3	0.11	14.81	0.57	63.90	0.75	61.74
	Vidyo4	0.20	16.29	1.04	65.36	1.04	63.96
Average		0.11	13.59	0.96	55.79	0.80	50.96

**Table 4:** Performance comparison of different parts of the proposed method (low delay (LD)).

		CTU Depth Decision		PU Mode Decision		Overall (Proposed)	
Size	Sequence	BDBR(%)	TS(%)	BDBR(%)	TS(%)	BDBR(%)	TS(%)
2560×1600	Traffic	0.12	9.34	0.92	54.54	0.89	54.81
	SteamLocomotive	−0.19	11.65	0.33	51.62	0.29	51.84
1920×1080	ParkScene	0.11	10.15	1.07	52.66	1.08	53.02
	Cactus	0.06	10.19	1.03	47.47	0.85	47.82
	BQTerrace	0.01	12.83	0.58	54.33	0.62	54.46
832×480	BasketballDrill	0.18	10.75	0.71	43.95	0.79	44.22
	BQMall	0.42	8.09	0.93	51.06	0.86	49.39
	PartyScene	0.22	9.16	0.58	40.92	0.55	41.29
	RaceHorses	0.02	6.73	0.79	37.04	0.89	37.03
416×240	BasketballPass	0.94	6.99	0.91	51.21	0.91	51.80
	BQSquare	0.10	4.33	0.54	44.85	0.36	42.24
	BlowingBubbles	0.24	3.92	1.16	40.16	1.15	40.57
1280×720	Vidyo1	0.18	20.30	0.68	64.11	0.70	64.13
	Vidyo3	0.25	12.33	1.04	58.55	1.01	58.95
	Vidyo4	−0.37	14.03	0.55	61.67	0.48	61.95
Average		0.15	14.05	0.79	50.28	0.76	50.23

**Table 5:** Performance comparison of different γ.

	(BDBR, TS)		
	γ = 0.5	γ = 0.75 (Proposed)	γ = 0.85
Random Access	(1.01, 55.25)	(0.80, 50.96)	(0.98, 51.83)
Low Delay	(0.86, 50.63)	(0.76, 50.23)	(0.82, 50.55)

**Table 6:** Performance comparison of the proposed method compared to previous works.

	Method	(BDBR, TS)
RA	Proposed	(0.80, 50.96)
	Zhang’s [12]	(1.19, 54.93)
	Zhu’s [13]	(3.67, 65.60)
	Ahn’s [14]	(1.40, 49.60)
	Goswami’s [24]	(1.11, 51.68)
	Tai’s [25]	(1.41, 45.70)
	Xiong’s [26]	(2.00, 58.40)
LD	Proposed	(0.76, 50.23)
	Zhu’s [13]	(3.84, 67.30)
	Ahn’s [14]	(1.00, 42.70)
	Tai’s [25]	(0.75, 37.90)
	Xiong’s [26]	(1.61, 52.00)
